# Effect of R Angle of the Outer Extension Tube against the in-Core Flux Thimble in Nuclear Power Plant on Its Wear Behavior

**DOI:** 10.1155/2021/1469642

**Published:** 2021-09-23

**Authors:** Qiang Chen, Yinhui Che, Jianjun Guan, Yang Li, Qinhu Wang

**Affiliations:** Suzhou Nuclear Power Research Institute Co., Ltd., Shenzhen 518120, China

## Abstract

Wear failure of the in-core flux thimble is an important problem in the neutron flux measurement system, which threatens the safety of the nuclear power plant. To figure out the wear mechanism of the thimble, a wear tester was designed and manufactured to simulate the wear process of the in-core flux thimble. Outer guide tubes with different R angles were used to abrade the thimbles. The designed tester can well simulate the wear process in the real power plant. R angle of the outer guide tube played important role in the wear behavior of the in-core flux thimbles.

## 1. Introduction

Nowadays safety of the nuclear power plant becomes more and more important along a big number of nuclear power plants go into operation. The flow around the thimbles of the in core instrumentation induces vibration and shocks against their guides in the vessel, producing wear and even leakage of the thimbles [1]. Almost all nuclear power plants in China were concerned by this problem seriously [2–4].

Although this problem has been noticed to be quite important, no study was focused on it till now. Impact and abrasion between the thimbles and the guide tubes is a very unique wear type, which consists of longitudinal shift and circular movement. No commercial device can be obtained to complete this kind of wear test. In order to characterize the phenomenon and find a way to reduce or suppress wear of the thimbles, a dedicated device have been designed and developed, which can simulate the relative movement between the thimbles and guide tubes.

The most serious wear was found to occur at the end of the outer guide tube with sharp shape. Changing the R angle of this end may help to control the damage of the thimble. So, one end of the outer guide tubes was manufactured as different R angles. These guide tubes were used to contact the thimbles by using the self-made device mentioned above.

## 2. Experimental

### 2.1. Test Device

**Figure 1:**
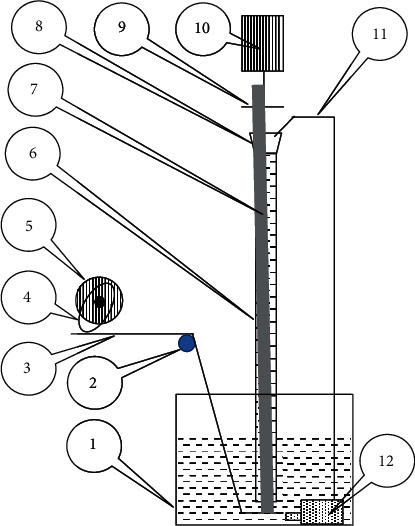
Diagram of the self-made device to simulate the wear of the thimbles. 1. Container; 2. Bearing; 3. Lever; 4. Cam; 5. Motor I; 6. Guide tube; 7. Thimble; 8. Funnel; 9. Stick; 10. Motor II; 11. Water tube; 12. Water pump.

As mentioned above, a dedicated device is needed to simulate the relative movement between the thimbles and guide tubes. The diagram of the device is shown in Figure 1.

The device contains a container for the water which supplies water for the space between the thimble and the guide tube by a water pump. A lever supported by a bearing can make the thimble up and down by a cam with motor I. A funnel is applied for decreasing splash of the water. A slim stick with motor II is used to drive the thimble to move circumferentially. Both motors are controlled by frequency converters which can change their rotational speed.

### 2.2. Experimental Parameters

The frequency of moving up and down was set as 5 Hz and the driving distance of the level was 5 mm. The rotation frequency of the thimble was set as 10 Hz. The circulating water can fill up the guide tube during the whole test period of 72 hrs. The R angle of the guide tubes were manufactured as R6.3 and R0, respectively.

The thimbles were made of Z5CND17-12. Its nominal chemical composition is shown in Table 1. The guide tubes were made of Z2CN19-10NS, whose nominal chemical composition is shown in Table 2.

### 2.3. Roughness and Profile Measurement

After the 72-hr test, profiles of the wear scars were measured by a roughness tester (Type 2300A-R, Harbin Measuring & Cutting Tool Group Co. Ltd.) to obtain the roughness and a KEYENCE VR-3200 3D profile tester to obtain depth of the scars.

### 2.4. SEM and EDS Analysis

The details of the wear scars were analyzed by using an FEI Co. XL30 ESEM FEG scanning electron microscope (SEM) with Energy Dispersive Spectrometer (EDS) to get the morphologies and surface composition of the scars.

### 2.5. Micro Hardness Measurement

Micro hardness of the original surface and the wear scars were measured by using a HVS-1000 M micro hardness tester produced by Shanghai Microcre Light-Mac. Tech. Co. Ltd. The load was 200 g and the holding pressure time was 10 s.

## 3. Results and Discussion

### 3.1. Macro Morphology of the Wear Scars

**Figure 2:**
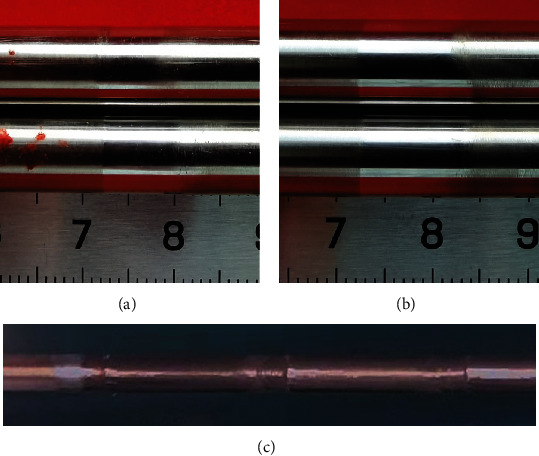
Macro morphology of the wear scars. (a) R0 (b) R6.3 (c) From nuclear power plant.

Figure 2 shows the macro morphologies of the wear scars on the thimble after 72 h of test (Figures 2(a), 2(b)), which are similar to the scars formed in the nuclear power plants (Figures 2(c)). The scars looked shiny like a ring around the thimble. These results confirmed the success of the test device which was designed and manufactured originally. Although the driving distance is the same as 5 mm in the test process, the scar width of the samples against the R6.3 is bigger than that of the R0. This means the guide tube with sharp R angle (0) can hold back the running distance of the sample more than that for the big R angle (6.3). Due to the inertia of the movement, the wear scar is wider than 5 mm.

### 3.2. Wear Depth and Wear Roughness

**Figure 3:**
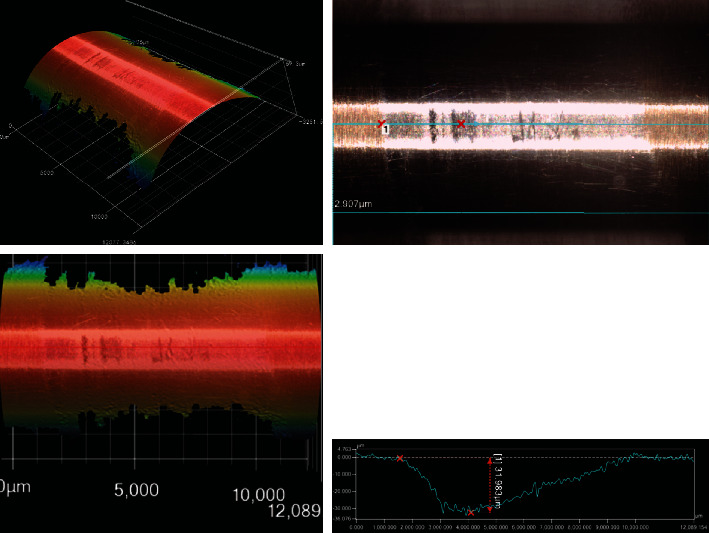
The typical profile of the wear scar and its maximum depth.

Six points near the upper end of the scar to measure the maximum depth based on the profile obtained by using the KEYENCE VR-3200 3D profile tester for each scar. The typical profile of the wear scar is shown in Figure 3 with the measurement of the maximum depth.

**Figure 4:**
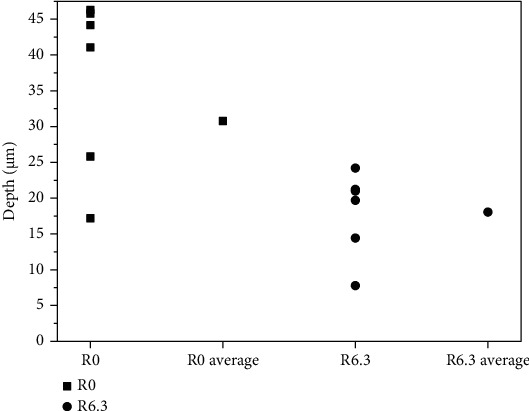
Maximum depth of the scars for the samples.

The average maximum depth for the sample against R0 is about twice of that for the sample against R6.3 as shown in Figure 4. Enlargement of the R angle for the guide tube can decrease the wear damage caused by the contact and abrasion between the thimble and the guide tube. The sharp angle (R0) of the guide tube can cut the thimble deeply and form sharp overhang in the horizontal direction. At the same time the sharp angle scrapes the thimble in the vertical direction thus remove some of the overhang easily. While the round angle (R6.3) of the guide tube cuts the thimble shallower due to its geometrical shape. In the vertical direction, the round angle squeezes the thimble instead of scraping for the sharp angle thus it is difficult to remove the material from the thimble. So the depth of the scars formed against the round angle was much smaller than that against sharp angle, indicating less material loss of the thimble.

**Figure 5:**
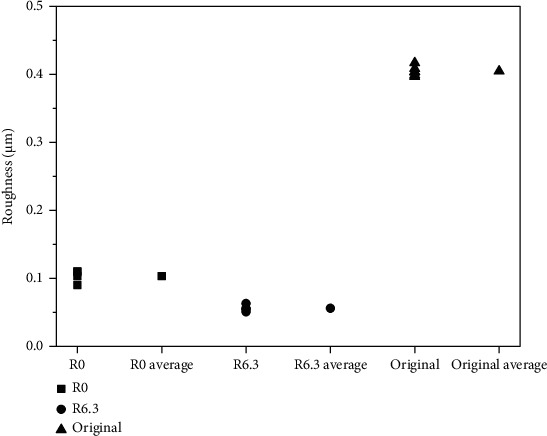
Roughness of the scars and the original surface.

The scar looks shiny and smooth compared to the original surface of the thimble. Roughness of the scars and the original surface was measured for six times and their average value was calculated as shown in Figure 5. Roughness in the scars is much smaller than that for the original surface of the thimble. Roughness for both R angles decreased significantly after the tests. Roughness for the sample against round R angle (6.3 mm) was the smallest which is about 60% of that for sharp R angle (0). When the R angle of the thimble is 0, the sharp angle of the guide tube can cut the thimble deeply in the horizontal direction. At the same time the sharp angle scrapes the thimble in the vertical direction. It is reasonable to say that the round angle (R6.3) of the guide tube cuts the thimble shallower due to its geometrical shape. In the vertical direction, the round angle squeezes the thimble instead of scraping for the sharp angle thus decreases the roughness. That's why the roughness of the scars formed against the round angle was much smaller than that against sharp angle.

Surface roughness always plays important role in the wear process of materials [5–11]. The most common conclusion of these works is that the bigger the roughness is, the more serious the wear damage is. This is an important reason why the wear depth of the thimble for the R0 guide tube is bigger than that for R6.3 (Figure 4). It is reasonable to predict that the thimble for the R0 guide tube will fail first if the test period is long enough.

### 3.3. SEM Morphology and EDS of the Wear Scars

**Figure 6:**
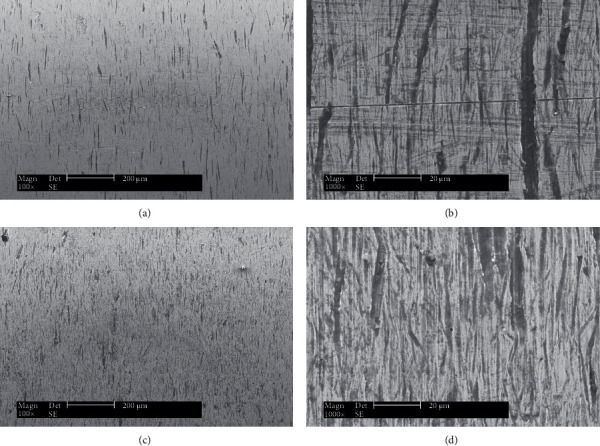
SEM morphologies of the scars against guide tube of R0 (a, b) and R6.3 (c, d).

SEM morphologies of the wear thimble scars of are shown in Figure 6. For both R angles short ploughings can be found around the surface of the thimble in the horizontal direction. Ploughings for the sample against sharp R angle (0) are fewer but deeper compared to that for the samples against round R angle (6.3 mm). With the sharp R angle, the guide tube can cut the thimble to form deep ploughings in the horizontal direction (Figure 6 a, b). With the sharp R angle, the guide tube can seriously cut the protuberance around the plouging and the ploughing itself in the vertical direction of the ploughing thus the old ploughings vanished quickly. While with the round R angle, the guide tube cut the thimble to form relatively shalow ploughings in the horizontal direction (Figure 6 c, d). Although they were shallow, the guide tube with round R angle (R6.3) cannot cut the protuberance around the plouging and the ploughing itself seriously thus the old ploughings vanished slowly. The results are well in accordance with the results of scar depth and scar roughness mentioned above.

**Figure 7:**
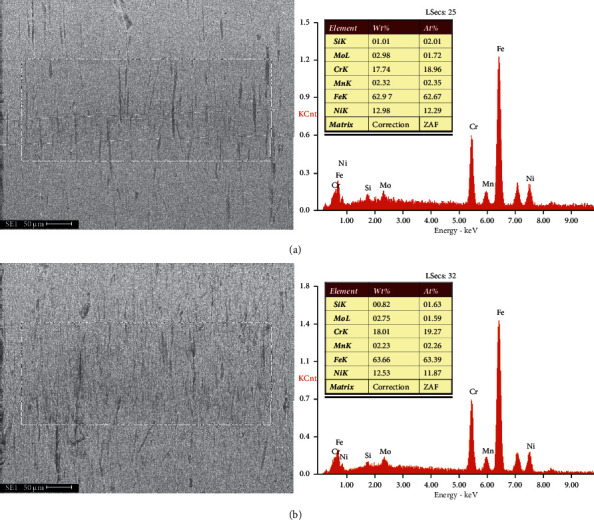
EDS of the scars against guide tube of R0 (a) and R6.3 (b).

For the EDS results (Figure 7), the chemical compositions in the scars were similar to the original alloy (Table 1). No apparent oxidation was found in both wear scars, indicating that the wear was mainly caused by mechanical damage while had nothing to do with chemical reactions.

### 3.4. Micro Hardness Measurement

**Figure 8:**
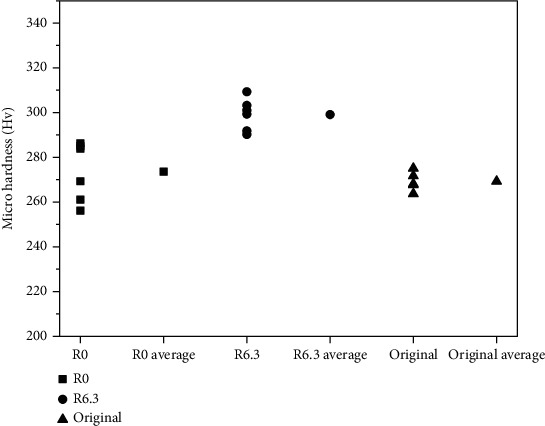
Micro hardness of the wear scars and the original surface.

Micro hardness of the wear scar formed by the R0 guide tube was almost the same as that of the original surface, while micro hardness of the wear scar formed by the R6.3 guide tube changed a lot (Figure 8). As mentioned above, deformation was found on the wear scar surface for both cases (Figure 6). Crossing slip dislocations formed during plastic deformation can create dipolar and multipolar structures that may contribute to work hardening. Such structures may become plastically relaxed by secondary slip thus the cross-slip of dislocation groups was an important work-hardening mechanism [12]. Z.Q.Wang et al. also found that cross-slip plays a role in the generation and annihilation of dislocations, leading to different dislocation velocities, density evolution and macroscale plastic response, which was related to work-hardening [13]. Although work-hardening occurred during the wear process, the hardness of the scar for the R0 guide tube changed little due to the fast removal of the surface layer.

### 3.5. Modeling the Impact and Abrasion of the Thimbles

**Figure 9:**
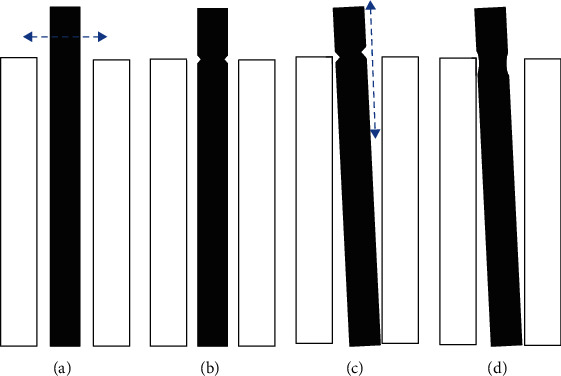
Schematic diagram of the wear process of the thimble against the guide tube.

Figure 9 shows a model for the impact and abrading process of the thimbles. When the thimble moves in horizontal direction in the guide tube (Figure 9(a)), it impacts the end of the guide tube and forms ploughings on the thimble surface (Figure 9(b)). When the thimble moves in vertical direction in the guide tube (Figure 9(c)), it is abraded by the end of the guide tube and cuts the ploughings formed on the thimble surface to make it shallow (Figure 9(d)) thus make the surface smoother. In fact, the movements of the thimble in horizontal and vertical direction occur simultaneously all the time. The ploughing and the abrasion caused the mass loss of the thimble and make it smoother, showing smaller roughness. The larger the R angle is, the shallower the ploughing is and the weaker the abrasion is, thus the less the mass loss is, showing smaller maximum depth of the wear scar.

## 4. Conclusion

1. The self-made device can well simulate the movement between the thimble and the guide tube and the wear process of the thimble in the nuclear power plant
2. Bigger R angle of the outer guide tube can decrease the roughness and increase the hardness of the wear scar more significantly which is beneficial to reduce wear of the thimble
3. R angle of the outer guide tube can significantly affect the wear behavior of the thimbles. Bigger R angle is beneficial to extending the service life of the thimbles

## Figures and Tables

**Table 1:** Nominal chemical composition of Z5CND17-12.

C	Mn	Si	P	S	Cr	Ni	Mo	Cu	Co	Nb + Ta
≤0.070	≤2.00	≤0.75	≤0.030	≤0.015	16.00-19.00	10.00-14.00	2.00-2.50	≤1.00	≤0.06	≤0.15

**Table 2:** Nominal chemical composition of Z2CN19-10NS.

C	Mn	Si	P	S	Cr	Ni	Cu	Co	Nb + Ta
≤0.035	≤2.00	≤1.00	≤0.030	≤0.015	18.50-20.00	9.00-10.00	≤1.00	≤0.06	≤0.15

## Data Availability

All data generated or analyzed during this study are included in this article.
